# In Vitro, Ex Vivo, and In Vivo Evidence of Nitrate-Reducing Activity in *Levilactobacillus brevis* CD2: A Potential Tool for Oral and Systemic Health Applications

**DOI:** 10.3390/foods14091512

**Published:** 2025-04-26

**Authors:** Serena Altamura, Francesca Rosaria Augello, Francesca Lombardi, Paola Palumbo, Benedetta Cinque, Davide Pietropaoli, Claudio De Simone

**Affiliations:** 1 Department of Life, Health & Environmental Sciences, University of L’Aquila, 67100 L’Aquila, Italy; serena.altamura@univaq.it (S.A.); francescarosaria.augello@univaq.it (F.R.A.); francesca.lombardi@univaq.it (F.L.); paola.palumbo@univaq.it (P.P.); benedetta.cinque@univaq.it (B.C.); 2 Department of Physical and Chemical Sciences, University of L’Aquila, 67100 L’Aquila, Italy; 3 Retired Professor of Infectious Diseases at the University of L’Aquila, 67100 L’Aquila, Italy; cds@ironmail.ch

**Keywords:** lactic acid bacteria, probiotics, *Levilactobacillus brevis* CD2, nitrate reductase, oral and systemic health

## Abstract

Growing evidence supports the use of nitrate-reducing bacterial strains as probiotics to enhance the benefits of nitrate metabolism for both oral and systemic health. This study aimed to test the nitrate reductase activity of *Levilactobacillus brevis* CD2 (DSM-27961/CNCM I-5566), a strain widely used as a starter culture in fermented foods and recognized for its multifaceted health-promoting probiotic properties. We also sought to determine whether the probiotic lysate enhances nitrate reduction ex vivo using six salivary samples from healthy subjects while evaluating its potential influence on pH and buffering capacity. Considering the established link between lactate metabolism and nitrite production, we assessed the salivary levels of D-lactate after a 3-hour incubation with or without *Lv. brevis*. The results indicate that *Lv. brevis* CD2 exhibits significant intrinsic and concentration-dependent nitrate reductase activity. Additionally, treatment with *Lv. brevis* for 3 h significantly increased nitrite generation across all saliva samples, with further enhancement observed after the addition of exogenous nitrates. *Lv. brevis* also significantly improved salivary pH and buffering capacity, particularly when combined with nitrate. Furthermore, the probiotic treatment resulted in reduced levels of salivary D-lactate. To further support and validate our in vitro and ex vivo findings, we evaluated the oral nitrate-reducing activity in saliva samples from healthy individuals treated for four weeks with *Lv. brevis* CD2 lozenges. Of note, the results indicated that the probiotic group showed a significant increase in oral nitrate-reducing capacity compared to baseline and placebo after four weeks of treatment. Overall, our study suggests that *Lv. brevis* CD2 acts as a nitrate-reducing probiotic, providing new insights into its health benefits and complementing findings from previous studies.

## 1. Introduction

Lactic acid bacteria (LAB) are a heterogeneous group of Gram-positive, catalase-negative, and non-spore-forming microorganisms that can be found in various environments, including plants, animals, and humans [1,2]. They are typically anaerobic or facultatively aerobic, exhibit acid tolerance, and primarily ferment substances, making them frequently used in food preservation and fermentation [3].

LAB, with the status Generally Recognized as Safe (GRAS), during fermentation, help inhibit the growth of pathogenic microorganisms by lowering pH levels through acid production, effectively preventing food spoilage [4,5,6]. In addition, LAB classified as GRAS are widely recognized as probiotics [7]. In recent years, there has been a growing emphasis on the safety processes of probiotic LAB for industrial applications, food supplements, and human consumption [8,9]. Safety assessments can differ based on regional regulations and guidelines. Several factors must be considered to ensure that probiotic LAB are deemed completely safe for human use, including dosage, the duration of toxicity studies, and the species of animals tested [9,10]. Furthermore, safe production procedures are crucial for the manufacturing of probiotic products. According to the International Scientific Association for Probiotics and Prebiotics, the goal is to establish a reliable safety evaluation system that takes into account the unique properties of probiotics, enabling their safe use in industrial settings [11]. Probiotic LAB, such as *Lactobacillus*, are frequently isolated from many fermented foods and are also found in the oral and gastrointestinal microbiomes, where they maintain a symbiotic relationship with their host [12,13]. Some reported benefits of LAB include promoting the growth of beneficial bacteria, inhibiting the adhesion of pathogenic microorganisms through competition for adhesion sites and co-aggregation, secreting antimicrobial molecules, modulating the immune system, and helping to maintain a balanced microbiome [14,15]. Certain LAB are noteworthy not only for their ability to produce lactic acid but also for synthesizing nitrate (NO_3_^−^) and nitrite (NO_2_^−^) reductase enzymes [16,17]. These enzymes facilitate the conversion of nitrates to nitrites and subsequently to nitric oxide (NO) [18,19,20]. The metabolism and degradation of nitrates and nitrites by LAB involve the action of these reductase enzymes along with acid degradation mechanisms. Additionally, LAB can utilize extracellular polysaccharides, peroxide-catalyzed oxidation, and antioxidant substances, such as polyphenols, to enhance their effectiveness in enzymatically reducing nitrites [20]. These processes can significantly lower the concentrations of biogenic amines in food products, inhibit the formation of N-nitrosamines, and further degrade N-nitrosamines, thus reducing their impact on human health and lowering the incidence of diseases, including cancer [6,21]. Evidence shows that nitrate-reducing bacteria are present in the human digestive system, including the mouth, esophagus, and gastrointestinal tract [19]. Numerous studies have highlighted the potential effects of these bacteria on human health and disease, particularly through various disease models [19]. This evidence suggests a promising approach for disease prevention and treatment using specific probiotics. In this context, there is a growing interest in utilizing specific probiotics as an alternative or adjunctive approach to promote both oral and systemic health. This is especially relevant because the oral cavity serves as the entry point to the respiratory system and the gastrointestinal tract, and it is directly connected to the bloodstream through highly vascularized oral tissues [22,23,24,25,26,27,28].

Specific LAB isolated from the oral cavity are particularly beneficial due to their ability to produce nitrite from dietary nitrate, which is thought to support systemic health [29,30,31,32,33].

Dietary nitrates are excreted into the oral cavity with saliva, where facultative anaerobic bacteria present in the mouth can convert them into nitrites. Once swallowed, nitrites are spontaneously converted into NO and other nitrogen oxides in the acidic environment of the stomach [34,35]. However, a small portion is also absorbed directly into the bloodstream, where it is transported to various tissues and reduces NO by hemoglobin and enzymes [36]. A plethora of studies indicates that increasing bioavailability of nitrates and nitrites from exogenous sources is associated with cardiovascular benefits, such as lowering blood pressure [37,38]. Notably, the antioxidants and polyphenols found in fruits and vegetables help prevent the formation of carcinogenic N-nitroso compounds from nitrite while simultaneously stimulating NO production [39]. In addition to systemic benefits, microbial reduction of nitrates in the mouth promotes oral health [32]. Oral bacteria, by reducing nitrate to nitrite and NO, inhibit the growth of sensitive species, such as anaerobes involved in periodontal diseases [40,41]. Nitrite production has also been shown to increase resilience against salivary acidification in vivo and in vitro, thus preventing caries development [32]. One potential mechanism for this is proton consumption during the denitrification or bacterial reduction of nitrites to ammonium. Additionally, lactic acid, which contributes to oral acidification, and hydrogen sulfide, a volatile compound involved in halitosis, can serve as electron donors in these processes [32]. Moreover, the bioavailability of nitrates and nitrites is significantly influenced by the human oral microbiome, which can affect the host by producing metabolites as well as modulating immune responses and gene expression [42]. Consequently, a decrease in nitrate-reducing bacteria in the mouth, along with an increase in pathogenic bacteria, may explain the correlation between poor oral health and systemic diseases [22,23]. Therefore, the targeted application of oral nitrate-reducing bacteria, combined with the consumption of nitrate-rich foods and vegetables, could serve as a practical and effective strategy to enhance NO production and maintain NO homeostasis in the body. This, in turn, may help alleviate disease symptoms and reduce the incidence and severity of certain conditions, offering a novel method for disease prevention and treatment [43].

*Levilactobacillus brevis* (*Lv. brevis*), formerly *Lactobacillus brevis* (*L. brevis*) [44], is widely used as a starter culture in the production of various fermented foods [45,46,47,48]. Moreover, its health-promoting features are well characterized and exploited as probiotic [49]. *Lv. brevis* CD2 (DSM-27961/CNCM I-5566) is an obligate heterofermentative LAB known for its oral probiotic properties that support various clinical applications. These properties include the ability to antagonize oral pathogens, inhibit or disrupt pathogen biofilms, colonize oral environments, and provide anti-inflammatory effects. Growing evidence from both in vitro and in vivo studies supports the use of *Lv. brevis* CD2 as an effective therapeutic adjuvant or alternative in oral medicine [50,51,52,53,54,55,56,57,58,59,60].

Building on the multiple benefits demonstrated by this probiotic, the present study aimed to determine *Lv. brevis*’s potential to reduce nitrates by measuring the level of nitrate reductase activity in vitro. We also evaluated the probiotic’s ability to reduce nitrates in vitro using salivary samples from healthy humans, both with baseline nitrate levels and after the addition of exogenous nitrates. Furthermore, we assessed the impact of *Lv. brevis* on salivary pH and its buffering capacity under the experimental conditions used. Moreover, we evaluated the levels of D-lactate in saliva after treatment with probiotic lysate, given the established link between lactate metabolism and nitrite generation. The in vitro and ex vivo results were confirmed by the in vivo study. The treatment with lozenges containing live *Lv. brevis* CD2 for one month significantly increased the nitrate reduction capacity in salivary samples from healthy subjects compared to those who received a placebo.

## 2. Materials and Methods

### 2.1. Preparation of Bacterial Lysate

The probiotic suspension was prepared by firstly dissolving each Mucomixx lozenge (Lot. N° 2301401; EOS2021 Srl, Rome, Italy) containing 10^9^ CFU *Levilactobacillus brevis* CD2 DSM-27961/CNCM I-5566 in 5 mL of phosphate-buffered saline (PBS, EuroClone, West York, UK). The suspension was centrifuged at 8600× *g*, washed twice in PBS, resuspended in 5 mL of PBS, and sonicated using a Vibracell sonicator (Sonic and Materials, Danbury, CT, USA). The sample’s absorbance was measured at 590 nm (Eppendorf, Hamburg, Germany) before and after each sonication step to verify the bacterial cell disruption. Total protein content was evaluated using a DC Protein Assay (Bio-Rad Laboratories, Hercules, CA, USA).

### 2.2. Nitrate-Reducing Activity in Lv. brevis CD2 Lysate

The nitrate reduction capacity in probiotic lysate was determined as previously described [61]. A 100 μL of 16 mM sodium nitrate (Sigma-Aldrich, St. Louis, MO, USA) dissolved in PBS was mixed with increasing amounts of the probiotic lysate, ranging from 0 to 6400 μg protein/mL. The total volume was then adjusted to 200 μL with PBS, resulting in a final sodium nitrate concentration of 8 mM. The samples were incubated in a thermomixer (Eppendorf, Hamburg, Germany) for 3 h at 37 °C and subsequently centrifuged at 14,000× *g.* The nitrite concentration in the supernatant was measured using the Griess reaction, as described below. In an additional set of experiments, the nitrate-reducing activity of *Lv. brevis* lysate at a concentration of 1600 μg protein/mL was evaluated after 3 h of incubation with varying substrate concentrations (ranging from 0 to 8 mM sodium nitrate). In the assays measuring enzyme activity, the nitrate reductase provided in the Nitrate/Nitrite Assay Kit (Sigma-Aldrich) was used as a positive control. A mixture of 10 μL of nitrate reductase and 10 μL of the co-factor solution was incubated under the experimental conditions described previously for 3 h at 37 °C. For comparison with the experimental probiotic, the enzyme activity was measured in micromoles of nitrite produced per minute (units of nitrate reductase).

### 2.3. Nitrate-Reducing Activity in Human Saliva Samples Ex Vivo

To evaluate the nitrate-reducing potential of *Lv. brevis* lysate ex vivo, we used frozen saliva samples that were previously collected from six healthy adults as part of a recently described clinical trial [60]. For the present study, saliva samples were taken from subjects aged 25 to 43 years old (n = 6, female = 4, male = 2) with permanent dentition and free from oral and systemic diseases. For all aspects relating to the clinical trial, please refer to Section 2.8. 

### 2.4. Salivary Nitrate Reduction Test

The nitrate-reducing capacity in saliva samples was determined by incubating them in a thermomixer for 3 h at 37 °C, either in the presence or absence of 8 mM nitrate, as previously described [62]. For the assay, 25 μL of water containing 80 mM sodium nitrate (Sigma-Aldrich) was added to 225 μL of saliva, with or without probiotic lysate at the concentration of 1600 μg protein/mL. The concentration of accumulated nitrites was determined as described below.

### 2.5. Nitrite Level Assay

Nitrite levels were measured using the Griess reaction with a nitrite assay kit from Sigma-Aldrich. Specifically, nitrite levels were assessed by applying the samples (either probiotic lysates or salivary samples treated as described above) to a 96-well microtiter plate, following the manufacturer’s instructions. The absorbance was measured by spectrophotometric reading at 550 nm using a microplate reader (Bio-Rad Laboratories). The nitrite content of each sample was evaluated based on a standard curve obtained by linear regression made with sodium nitrite solution and expressed in µM.

### 2.6. pH Measurements and Buffering Capacity of Saliva Samples

The pH measurements were conducted using a digital pH meter (Mettler Toledo, Columbus, OH, USA) both before and after a 3-hour in vitro incubation to assess nitrate-reducing capacity, as described above. To evaluate the buffering capacity of saliva samples, the Ericsson method was employed [63]. For this assessment, 0.5 mL of saliva was combined with 1.5 mL of 5 mM hydrochloric acid (HCl) both before and after the 3-hour incubation with different treatments. The mixture was vigorously shaken (Corning Incorporated, Corning, NY, USA) and allowed to stand for 10 min. Lastly, the pH was recorded using the digital pH meter.

### 2.7. Salivary D-Lactate Levels

Salivary D-lactate levels were measured using a D-lactic acid/lactate colorimetric assay kit (Immunological Sciences, Rome, Italy), according to the manufacturer’s instructions. The absorbance was assessed using spectrophotometric reading at 530 nm (microplate reader; Bio-Rad Laboratories). The D-lactate concentration was calculated according to the standard curve values and expressed in µM.

### 2.8. In Vivo Study Design

The potential influence of *Lv. brevis* CD2 in vivo on oral nitrate-reducing capacity was evaluated on saliva samples collected from subjects enrolled for a clinical trial approved by the Internal Review Board (Protocol No. 48/2022, 22 November 2022) of the University of L’Aquila (Italy) and registered on the clinicaltrials.gov registry with registration number NCT06457724 (7 June 2024). The original clinical trial design, protocol, randomization, blinding, participant eligibility, and intervention were described in a previous publication of our group, reporting a first tranche of data [60]. The research received no external funding, and the trial was designed following the ethical principles of Helsinki’s Declaration for Human Clinical Studies [64]. Written informed consent was obtained from all subjects that participated in this study and the data were published for research and educational purposes. As described, the trial involved 30 healthy subjects that were double-blinded and randomly assigned to either the probiotic interventional group (n = 15, 7 females; 8 males; aged 37.93 ± 14.16 yrs) or the placebo control group (n = 15, 7 females; 8 males; aged 38.13 ± 13.21 yrs). Mucomixx lozenges (EOS2021 Srl, Lot. N° 2301401) containing probiotic *Lv. brevis* CD2 were used in the in vivo study. The placebo lozenges had the same composition as the probiotic, except for the active bacterial component. Specifically, the supplements contained sorbitol, hydroxypropyl cellulose, xylitol, magnesium stearate, talc, silicon dioxide, and artificial strawberry flavoring. All participants took four lozenges daily for four weeks. No adverse events were observed throughout the course of this study.

### 2.9. Whole-Mouth Nitrate Reduction

Participants were instructed to hold 10 mL of water containing sodium nitrate (80 µM) in their mouth for 5 min, as previously described [65]. The mouth rinse was then collected into a Falcon sterile tube and centrifuged at 2150× *g* for 10 min at 4 °C. The supernatant was collected and stored at −80 °C before measurement of absolute nitrite concentration with the above-described assay. The oral nitrate reduction capacity was evaluated in both the placebo and probiotic groups at different time points—before treatment (T0), at the end of treatment (T1), and following a two-week washout period (T2).

### 2.10. Statistical Analysis

Statistical analysis was performed using GraphPad Prism version 8.02 (GraphPad Software, San Diego, CA, USA). Data normality was assessed using the Shapiro–Wilk test. Non-normally distributed data were compared using a two-sided paired Wilcoxon signed-rank test (within-group comparison) or a two-sided Mann–Whitney test (between-group comparison). Oral nitrate-reducing capacity in saliva samples from the *Lv. brevis* and placebo groups was analyzed using a two-way repeated measures analysis of variance (ANOVA) test followed by Bonferroni post hoc test. Data were expressed as means ± SD or SE, or median and interquartile range (IQR, 25th–75th percentile) as specified in figure legends. Furthermore, *p*-values were considered statistically significant when lower than 0.05. To plot a heat map of different parameters evaluated in saliva samples after incubation with or without *Lv. brevis* CD2 for 3 h, data were normalized by z-score transformation.

## 3. Results

### 3.1. Nitrate-Reducing Activity in Lv. brevis CD2 Lysate

**Figure 1:**
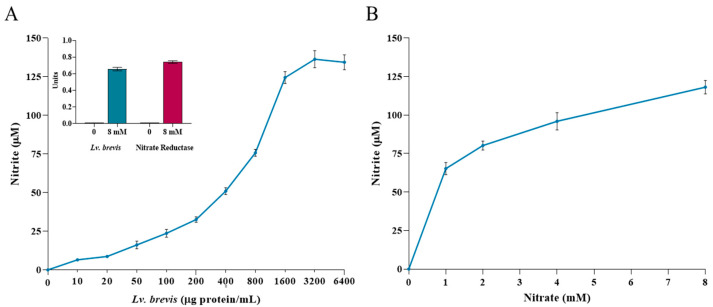
Nitrate reductase activity of *Lv. brevis* CD2 lysate. (**A**) Probiotic lysate was incubated at various concentrations (μg protein/mL) with an 8 mM nitrate solution for 3 h at 37 °C. The nitrite concentration in the supernatant was measured using the Griess reaction. The results are expressed as mean ± SE of three independent experiments performed in triplicate. The insert summarizes the activity of nitrate reductase used as an internal positive control as compared to that of the probiotic at 1600 μg protein/mL. (**B**) The nitrate reductase activity of *Lv. brevis* lysate, evaluated at a concentration of 1600 µg protein/mL, was assessed using different substrate concentrations ranging from 1 to 8 mM of sodium nitrate. The values are expressed as mean ± SE of three independent experiments performed in triplicate.

The primary objective of this study was to evaluate the effectiveness of *Lv. brevis* lysate in reducing nitrates to nitrites in vitro. As shown in Figure 1A, *Lv. brevis* lysate successfully reduced nitrates to nitrites in a concentration-dependent manner. The nitrate-reducing activity, expressed in µM, appeared to be closely related to the protein concentration of the probiotic added to the reaction system, with a proportional increase in enzyme activity. The results indicated that at concentrations exceeding 1600 μg protein/mL of *Lv. brevis*, the production of nitrites did not change substantially. Therefore, we chose to use 1600 μg protein/mL for the subsequent experiments involving salivary samples. The use of nitrate reductase as an internal positive control resulted in nitrite production of 133.14 ± 2.74 μM when 8 mM nitrate was used as the substrate. This result was comparable to that of the probiotic at higher concentrations. The insert in Figure 1A summarizes the enzyme activity results expressed in units (micromoles of nitrites produced per minute). Additionally, the reduction in nitrates and the subsequent generation of nitrites depended on the nitrate substrate concentration, as shown in Figure 1B. This finding further supports the specificity of the enzymatic reaction, as also confirmed by the observation that nitrite generation was not detected in the absence of nitrate.

### 3.2. Nitrate-Reducing Activity of Lv. brevis CD2 in Human Salivary Samples

**Figure 2:**
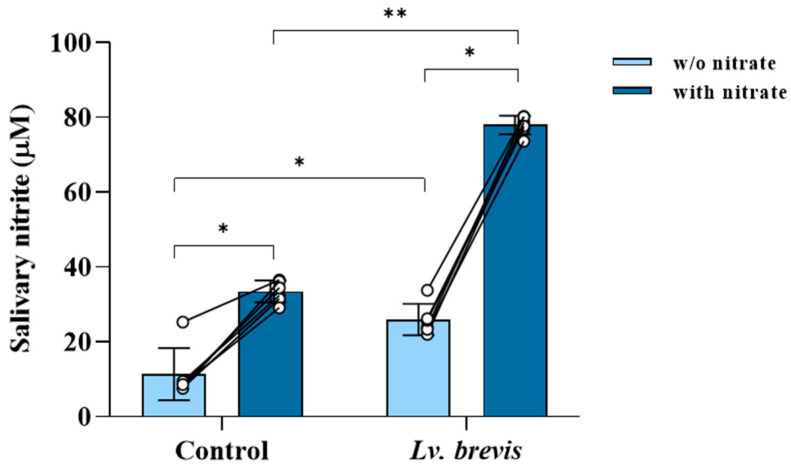
Nitrate reduction activity of *Lv. brevis* CD2 in saliva samples from healthy human subjects. The bar graphs represent the concentration of nitrite in saliva samples that were incubated ex vivo for 3 h at 37 °C without (control) or with nitrate solution (8 mM). Where indicated, the saliva samples were treated with *Lv. brevis* CD2 lysate (1600 μg protein/mL) with or without the nitrate solution. The circles represent the individual data, while the connecting lines show the changes in each sample with or without nitrate. The values are expressed as mean ± SD of one representative experiment performed in triplicate from three independent experiments. Within-group comparisons were performed using a two-sided paired Wilcoxon signed-rank test, and between-group comparisons were performed using a two-sided Mann–Whitney test (* p < 0.05; ** *p* < 0.01).

The *Lv. brevis* lysate was assessed in vitro for its ability to enzymatically reduce nitrates in saliva samples from six healthy subjects. The baseline nitrate-reducing activity in all salivary samples, measured by nitrite levels using the Griess reaction, ranged from 7.69 to 25.4 µM (Figure 2). As expected, the addition of an exogenous nitrate solution (8 mM) significantly enhanced the activity of salivary nitrate reductase across all samples, promoting nitrate reduction. Notably, the addition of *Lv. brevis* lysate resulted in a substantial increase in nitrate reduction in all six salivary samples compared to the untreated controls. The effect of *Lv. brevis* was comparable to that of the exogenous nitrate alone. The combination of the probiotic lysate and the exogenous nitrate solution produced a significant increase in nitrite levels compared to either treatment alone.

### 3.3. Effect of Lv. brevis CD2 on Salivary pH and Buffering Capacity

**Figure 3:**
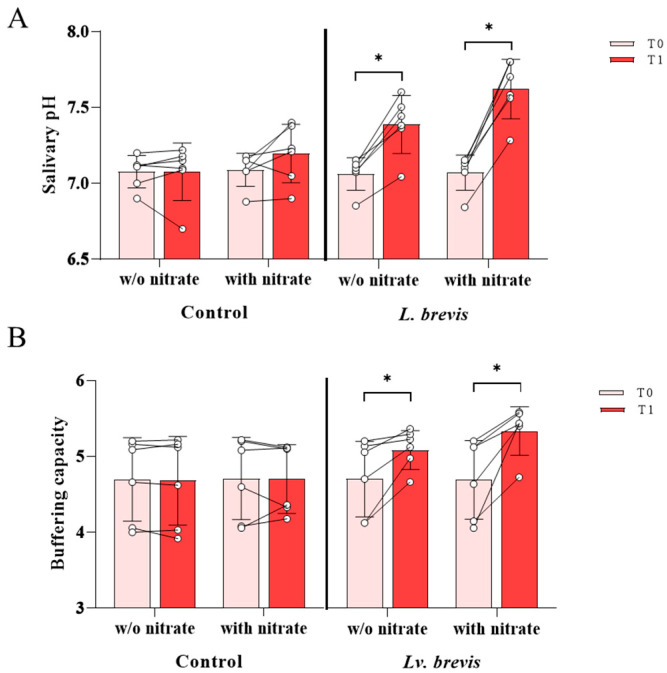
Effect of *Lv. brevis* CD2 on pH levels of salivary samples at T0 and after 3-hour incubation (T1), both with and without the addition of exogenous nitrates (8 mM) (Panel (**A**)). Effect of *Lv. brevis* CD2 on buffering capacity of salivary samples at T0 and after 3-hour incubation (T1) in the presence or absence of exogenous nitrates (8 mM) (Panel (**B**)). The circles represent the individual data, while the connecting lines show the changes in each sample before (T0) and after (T1) incubation. All the values are expressed as mean ± SD from one representative experiment performed in triplicate of three independent experiments. Within-group comparisons were performed using a two-sided paired Wilcoxon signed-rank test and between-group comparisons using a two-sided Mann–Whitney test (* *p* < 0.05).

As expected, in control untreated samples, no significant changes in salivary pH values were detected after 3 h of incubation at 37 °C (T1) compared to baseline (T0) (Figure 3A). On the other hand, the incubation with *Lv. brevis* CD2 significantly increased the salivary pH compared to T0, both in the presence or absence of exogenous nitrates (*p* < 0.05) (Figure 3A). Regarding the differences between the groups, the saliva samples treated with *Lv. brevis* CD2 exhibited significantly higher pH values as compared to relative untreated controls (*p* < 0.05). The combined treatment of probiotics with exogenous nitrate induced a more relevant increase in pH values than relative controls (*p* < 0.01).

After a 3-hour incubation with *Lv. brevis* CD2 (T1), the buffering capacity significantly increased compared to baseline (T0) both with and without exogenous nitrates (*p* < 0.05) (Figure 3B). The untreated control samples showed no significant changes in buffering capacity at T1 compared to T0 (Figure 3B). In terms of differences between the groups, the buffering capacity of saliva samples treated with *Lv. brevis* CD2 increased as relative to untreated controls, although this increment was not statistically significant. In contrast, the combined treatment of probiotic with exogenous nitrate resulted in a more pronounced increase in buffering capacity compared to the relative control (*p* < 0.05).

### 3.4. Effect of Lv. brevis CD2 on D-Lactate Salivary Levels

**Figure 4:**
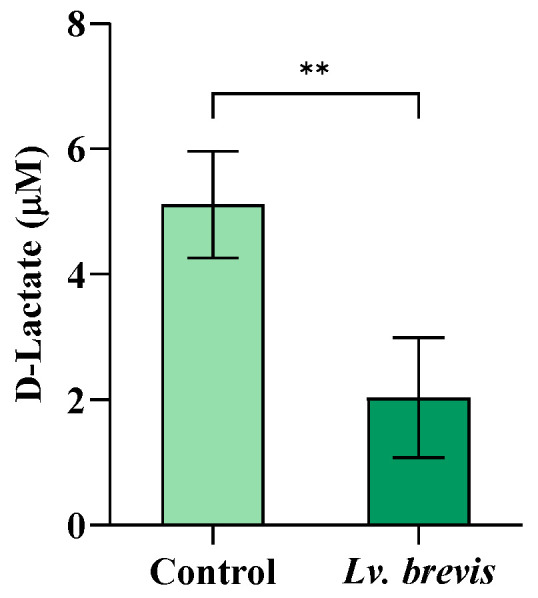
Effect of *Lv. brevis* CD2 on D-lactate levels in human salivary samples after 3-hour incubation. The values are expressed as mean ± SD of one representative experiment performed in triplicate from three independent experiments. Between-group comparison was performed using a two-sided Mann–Whitney test (** *p* < 0.01).

Considering the relationship between lactate metabolism and nitrite production, lactate being an electron donor in the nitrate reduction process, we measured the levels of D-lactate in saliva samples ex vivo, before and after a 3-hour incubation (T0 and T1, respectively) with or without *Lv. brevis* lysate at 1600 µg protein/mL. Our results showed that treatment with probiotics led to a relevant and statistically significant reduction in D-lactate levels compared to the control (*p* < 0.01) (Figure 4).

**Figure 5:**
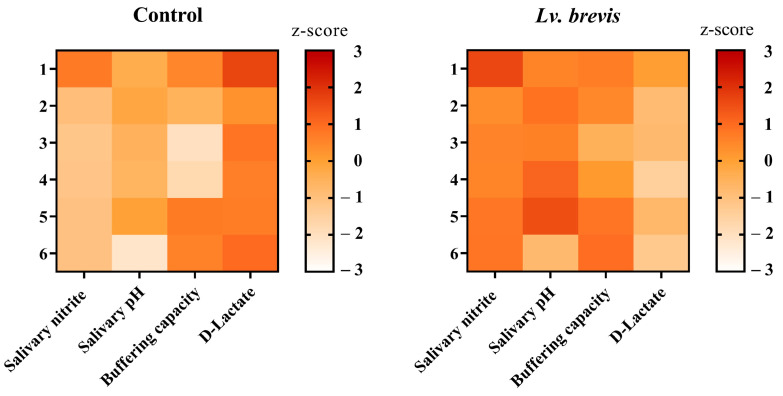
Heatmap illustrating the z-scores of the parameters evaluated in saliva samples incubated with or without *Lv. brevis* CD2 for 3 h. Darker shades indicate higher values, while lighter shades represent lower values. The left heatmap displays the untreated saliva samples (control), while the right heatmap represents the saliva samples treated with *Lv. brevis* CD2.

The heatmaps presented in Figure 5 illustrate the values of salivary parameters of all samples included (control and *Lv*. *brevis* CD2 treatment) at T1. Darker shades indicate higher values, while lighter shades represent lower values. After probiotic treatment at the individual level, the z-scores highlight an evident improvement in all the evaluated salivary parameters compared to the untreated saliva samples.

### 3.5. Effects of Lv. brevis CD2-Containing Lozenges on Oral Nitrate-Reducing Capacity In Vivo

**Figure 6:**
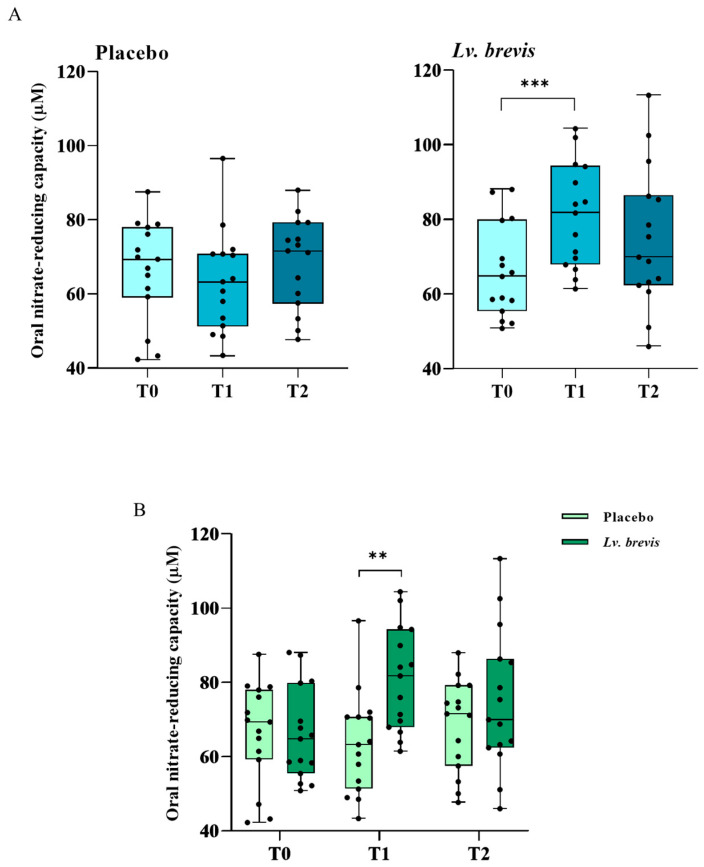
Evaluation of oral nitrate-reducing capacity assessed by nitrite levels (µM) in the placebo and *Lv. brevis* CD2 groups at T0, T1, and T2 (Panel (**A**)) (*** p < 0.005). Panel (**B**) shows the differences between placebo and probiotic groups at different times (** *p* < 0.01). Boxplots show the median and interquartile range (IQR, 25th–75th percentile).

The four-week treatment with *Lv. brevis* CD2 resulted in a highly significant increase in oral nitrate reduction capacity compared to baseline (*p* < 0.005) (Figure 6). These elevated values remained consistent after the washout period when compared to baseline, although the difference was no longer statistically significant (Figure 6A). In contrast, the placebo group showed no significant changes after the treatment or during the washout period. When comparing the two groups, the intake of *Lv. brevis* CD2 significantly enhanced nitrate-reducing potential both after treatment and following the washout, although the difference was statistically significant only at T1 (*p* < 0.01) (Figure 6B).

## 4. Discussion

In our current study, we examined the nitrate-reducing potential of the probiotic *Lv. brevis* CD2, which belongs to the LAB group and is known for its numerous beneficial properties, particularly in oral medicine. Our findings indicate that *Lv. brevis* CD2 exhibits significant concentration-dependent intrinsic activity of nitrate reductase. This activity was also observed when the probiotic was added to saliva samples from healthy adults, and it notably increased with the addition of exogenous nitrates. Based on these preliminary results, *Lv. brevis* CD2 may enhance the production and bioavailability of nitrate-derived products.

Another key objective of the present study was to investigate the ability of the probiotic *Lv. brevis* CD2 to positively affect salivary pH, a crucial factor for maintaining oral health and preventing dental erosion, as well as the formation of harmful biofilms [66]. Indeed, low salivary pH promotes the growth of acid-producing bacteria, which creates an unfavorable environment for protective oral bacteria [67]. Interestingly, while the baseline pH values of the saliva samples were within the normal range, treatment with the probiotic significantly increased the pH of each sample. This indicates that the nitrate-reducing activity led to an increase in salivary pH, a condition that helps inhibit the acid-producing capabilities of bacteria associated with dental caries [68]. This nitrate-dependent mechanism, along with the arginolytic properties of the *Lv. brevis* CD2 strain that generates ammonia ions, could help maintain the physiological pH level of saliva [51,69,70,71]. Furthermore, the increase in pH was significantly greater when *Lv. brevis* CD2 was combined with exogenous nitrate compared to when each treatment was applied individually, thus confirming the important role played by nitrate reduction in increasing saliva pH. Our results indicate that also the buffering capacity of saliva samples treated with the probiotic significantly improved when compared to untreated samples. Saliva’s buffering capacity is an important factor because it helps maintain pH levels, neutralizes acid exposure, and prevents enamel demineralization and dental caries [72]. These findings align with our recent data showing that *Lv. brevis* CD2 effectively protects dental enamel from damage caused by demineralizing agents and enhances the enamel’s resistance to demineralization in vitro [59]. Thus, the nitrate-reducing capacity of CD2 may provide an additional mechanism for preventing acidification in the oral cavity, potentially helping to prevent oral diseases such as tooth decay.

The current study explores an intriguing concept regarding the relationship between the nitrate reduction capacity of *Lv. brevis* CD2 and the increase in salivary pH and buffering potential. The combination of these two factors may create a synergistic mechanism that could help maintain oral health. Specifically, the nitrate reduction pathway may rise salivary pH by utilizing lactate (and other electron donors) to convert nitrate into nitrite, which in turn reduces the accumulation of acidic byproducts in the oral environment. As a result, this could not only suppress acid-producing bacteria but also foster a more favorable environment for protective oral microbes [73,74]. Additionally, the oral microbiome consists of multiple metabolic networks that can intersect with the nitrate reduction pathway. For instance, the arginine deiminase (ADI) system and urea catabolism release ammonia, which neutralizes acids and consequently increases oral pH [75,76]. It is plausible that *Lv. brevis*, by reducing nitrates, may synergistically interact with other pathways of acid neutralization, collectively enhancing the buffering capacity of the oral environment. Future research will help determine whether the co-administration of specific strains of LAB, along with diets rich in arginine or nitrates, has a combined or additive effect on stabilizing salivary pH and promoting overall oral homeostasis.

An important extension of this study is the investigation of the effects of *Lv. brevis* CD2 on salivary D-lactate. While many studies on lactic acid bacteria (LAB) focus on L-lactate metabolism, D-lactate metabolism has distinct clinical and inflammatory implications [77]. The accumulation of D-lactate in the oral cavity and the gastrointestinal tract has been associated with dysbiosis and inflammatory responses, although precise causal links are still being explored [77,78,79]. Our experiments assessed the impact of probiotic treatment on salivary levels of D-lactate, a crucial component for oral health. We found a significant reduction in D-lactate in saliva samples treated with *Lv. brevis* CD2 compared to the untreated group. This finding supports our previous in vivo results obtained after one month of intake of lozenges containing the live probiotic [60]. Indeed, salivary D-lactate levels were significantly reduced in subjects treated with *Lv. brevis CD2* for one month compared to the baseline or placebo group, and the effect remained significant and stable even during follow-up after two weeks. This finding represents the first evidence that a probiotic can influence baseline salivary D-lactate levels. Our results, also corroborated by our previous in vivo results [60], indicate that *Lv. brevis* lowers D-lactate levels, highlighting a potentially important pathway that may confer clinical benefits in terms of reducing inflammatory or cariogenic risk. Additionally, our data suggest that the relationship between lactate consumption and the nitrate reduction pathway, previously observed in certain bacteria isolated from the oral microbiome [80,81], also applies to *Lv. brevis*.

Of particular interest are the results obtained from one month of treatment with *Lv. brevis* CD2 lozenges regarding oral nitrate-reducing capacity, which reinforce our ex vivo findings. These results suggest that bacterial enzymatic activity can function effectively in the oral environment, yielding beneficial effects on oral health. Although the benefits of probiotic use continued after treatment ended, these effects were not statistically significant. This could indicate that the treatment duration was too short or that ongoing use of the probiotic is necessary. Additionally, it is crucial to determine how treatment with *Lv. brevis* CD2 affects the oral microbiome. In this regard, ongoing research from the previously mentioned clinical trial is focused on key nitrate-reducing bacteria, including *Neisseria*, *Rothia*, *Actinomyces*, and *Kingella* [32,80]. The nitrate–nitrite–nitric oxide pathway relies on oral microbiota and can offer various cardiovascular and metabolic benefits when stimulated by dietary nitrate [29,82]. These benefits include lower blood pressure, improved endothelial function, reversal of metabolic syndrome, antidiabetic effects, and enhanced exercise performance in certain situations [33,82,83]. Overall, our findings are promising and suggest that *Lv. brevis* can be regarded as a suitable and safe probiotic candidate for maintaining or restoring healthy levels of nitrate reduction in the mouth. This applies to both preventive measures and pathological conditions. When using oral nitrate-reducing bacteria, it is essential to carefully consider the safety of the bacterial strains involved. Scientific reports emphasize the importance of this, as some bacteria with nitrate-reducing capabilities, such as *Staphylococcus aureus*, also have pathogenic properties [84]. However, further considerations are essential, which should lead to more in-depth studies in this important area for both oral and systemic health. Furthermore, it is advisable to monitor potential nitrosamine formation following the intake of oral probiotics with nitrate-reducing capabilities [85]. Additionally, some authors emphasize that using nitrate-reducing probiotics in combination with a balanced diet that includes nitrate supplementation is both safe and effective [86,87]. Thus, disease prevention and treatment could benefit from the targeted use of oral nitrate-reducing probiotics alongside the consumption of nitrate-rich foods. This approach may be both practical and effective for promoting NO production and maintaining NO homeostasis in the body. As a result, it may help alleviate disease symptoms and reduce the incidence and severity of various conditions, providing an innovative strategy for disease prevention and treatment.

## 5. Conclusions

To the best of our knowledge, this study provides the first evidence of nitrate-reducing activity in the *Lv. brevis* CD2 strain, highlighting its effectiveness both ex vivo and in vivo. Although further investigation is needed to achieve complete biochemical and molecular characterization of the enzymes responsible for the observed nitrate-reducing activity in *Lv. brevis* CD2, these findings propose a new mechanism that may explain the various beneficial effects of this probiotic on both oral and systemic health.

## Data Availability

The data that support the findings of this study are available upon reasonable request from the corresponding authors.
